# Emerging therapies and evolving paradigms in cardiovascular pharmacotherapy

**DOI:** 10.1093/ehjcvp/pvag053

**Published:** 2026-08-17

**Authors:** Bianca Rocca

**Affiliations:** Department of Medicine and Surgery, LUM University, Casamassima, Bari 70010, Italy

The antithrombotic management of residual cardiovascular risk post-acute coronary syndromes (ACS) and post-ischaemic non-cardioembolic stroke remains an unmet therapeutic need, despite major improvements in cardiovascular care and secondary prevention.^1^ Novel antithrombotic strategies, used alone or in combination, aimed at reducing recurrent thrombotic events face the challenge of improving efficacy without significantly impacting bleeding, especially in the months immediately following the acute event. In the current issue, Caso *et al*.^2^ revisit the pathophysiology of factor XIa and its inhibition as a novel anticoagulation strategy evaluated in recent trials ranging from primary prevention of cardioembolic stroke in atrial fibrillation (AF) to the secondary prevention of non-cardioembolic stroke on top of antiplatelet therapy. Factor XIa blockade has been developed under the hypothesis of uncoupling thrombosis from haemostasis, offering safer and still effective antithrombotic agents. The authors provide a highly translational overview, spanning coagulation, organ-specific thrombotic mechanisms, and results of recent phase III trials of factor XIa inhibition, which proved effective when added to antiplatelet therapy in secondary prevention after non-cardioembolic stroke but failed to match apixaban in AF.^2^

**Figure:**
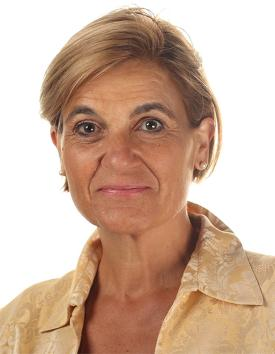


Post-operative AF (POAF) is a common, often transient complication after major surgery.^3^ Ravn and Milojevic^4^ unravel the complexity of the central therapeutic ‘dilemma’ of POAF occurring after heart surgery, between the need for thromboembolic protection and the risk of major bleeding. Their critical overview provides the framework for the large observational study from Rezk *et al*.,^5^ including 4436 patients who developed POAF after coronary artery bypass grafting (CABG) between 2009 and 2020, without oral anticoagulation at discharge. Guidelines suggest using anticoagulation for different time intervals after discharge based on the ‘bleeding risk’ of the individual patients, but without clear guidance and criteria to evaluate this risk. The authors performed a proof-of-concept study, testing the association of the four-item PRECISE-DAPT score (age, creatinine clearance, preoperative haemoglobin concentration, and previous bleeding) with major bleeding during the first post-operative year. The score showed moderate discrimination and reasonable calibration to identify patients who developed major bleeding, although its overall predictive performance remained modest. Whether this (and other) risk scores may help tailor anticoagulation in patients experiencing POAF post-CABG needs further study.

The continuing expansion of cardio-renal-metabolic pharmacotherapy represents another strategy to reduce cardiovascular risk. Semaglutide, originally approved as a glucose-lowering agent,^1^ is now an important treatment for obesity and for cardiovascular risk reduction.^6^ Using a target-trial emulation approach, Malik *et al*. investigated semaglutide in patients with type 2 diabetes mellitus (DM), obesity, and rheumatoid arthritis, a population with a particularly high cardiovascular risk profile, but without established atherosclerotic disease or symptomatic heart failure (HF). As highlighted by the PharmaPulse by Semb *et al*.,^7^ rheumatoid arthritis *per se* is associated with accelerated atherosclerosis and atherothrombosis, supporting the need for a dedicated ‘cardio-rheumatology’ multi-disciplinary approach. In this observational analysis based on the TriNetX database and non-glucagon-like peptide-1 receptor agonists (GLP-1 RAs) drugs as a comparator, semaglutide was associated with reduced major adverse cardiovascular and cerebrovascular events over approximately a 2-year follow-up, an association largely driven by fewer incident HF.^8^ Although observational in nature, this target-trial emulation suggests that semaglutide, and likely GLP1-RAs more broadly, may confer cardiovascular benefit in primary prevention for this very high-risk setting.

Extending the focus on residual cardio-renal-metabolic risk, Borghi *et al*. review serum uric acid as a biomarker and potential drug target in cardiovascular diseases. The authors conclude that, despite accumulating genetic and epidemiological evidence, routine urate-lowering therapy has no data supporting its use for cardiovascular prevention beyond approved indications in gout or symptomatic hyperuricaemia.^9^ Their review highlights the distinction between risk markers and validated therapeutic targets, a recurring challenge and source of biases in contemporary cardiovascular pharmacotherapy.

Beyond their established ability to reduce hospitalization and cardiovascular mortality,^10^ sodium–glucose cotransporter-2 (SGLT2) inhibitors may contribute to therapeutic simplification in patients with HF with reduced ejection fraction.^11^ Tomasoni *et al*.^11^ by using data from the Swedish Heart Failure Registry, suggest that initiation of SGLT2 inhibitors is associated with a sustained reduction in loop-diuretic requirements and fewer subsequent diuretic escalations. Finally, Drexel *et al*.^12^ focus on the impact of cardiovascular prevention through lipid-lowering drugs specifically in patients with HF, extending the series of lipid management in complex clinical settings, such as liver disease, type 1 and type 2 DM.^13–15^ The authors emphasize an important paradox: while lipid-lowering therapies substantially reduce the risk of developing HF by preventing atherosclerotic disease, their benefits become considerably less apparent once HF is established.

## Data Availability

No new data were generated or analysed in support of this research.
